# Fab five: pioneering sociocultural influence within the culture of basketball and American society

**DOI:** 10.3389/fspor.2024.1228440

**Published:** 2024-08-16

**Authors:** M. Fennell, C. K. Harrison, R. D. Manning, J. Boyd, O. Stuart, D. Scott, M. Martin, K. Dwyer, L. Proctor, S. Bukstein

**Affiliations:** ^1^ Sport Administration, University of Louisville, Louisville, KY, United States; ^2^ Sport Business Management, UCF, Orlando, FL, United States; ^3^ Sport Management, University of San Francisco, San Francisco, CA, United States; ^4^ Independent Researcher, Tempe, AZ, United States; ^5^ Independent Researcher, Watchung, NJ, United States; ^6^ Independent Researcher, Detroit, MI, United States; ^7^ Athletics, University of Missouri Kansas City, Kansas, MO, United States; ^8^ Sport Administration, Grambling State University, Grambling, LA, United States

**Keywords:** basketball, culture, hip hop, smack talk, disruption

## Abstract

Dating back to when the inventor of the game, James Naismith, developed a mentoring relationship with John McClendon one of the African American pioneers in basketball (founder of the “fast-break”), there are countless examples of these intersections. Entering the college basketball culture as the most decorated recruiting class in National Collegiate Athletic Association basketball history, the University of Michigan Fab Five's legacy catalyzes a new era of American basketball culture. Gracefully talented, the Fab Five abruptly disrupted the institution of basketball, the National Collegiate Athletic Association, and the identity of basketball athletes globally. This paper presents a sociocultural exploration of the residual impact of the Fab Five's legacy. As authentic, confident, and culturally competent, the five young men intentionally resisted and acknowledged the intersections of race, culture, and class within the college basketball culture. We critically assess the evolution of basketball culture, grounded by the sociocultural experiences of the Fab Five, imprinting upon contemporary generations of college basketball programs and their player. Through these experiences, the Fab Five's success through conflict, during their short stint in college basketball and beyond their professional careers trailblazed a path for the modern-day basketball athlete. Known for their style of play, their expression of fashion on and off the court, and eagerness to talk smack, the Fab Five backed up their talk with performance. Their performance on and off the court, revolutionized the culture of basketball; Even more, American society. The Fab Five's legacy is the cultural catalyst for basketball culture on all levels.

## Introduction to the Michigan Fab Five

Critically, the case of the 1991–1992 University of Michigan Men's Basketball team served as the foremost sociological intersection of culture, influence, and sport; more specifically, the intersection of black culture and basketball. Led by the pioneering true-freshmen known collectively as the Fab Five, the University of Michigan's program enabled athletes to feel more comfortable expressing themselves culturally both vocally and in their fashion. In particular, an emerging expression of hip-hip culture, wearing head phones, listening to and reciting hip-hop lyrics, wearing baggy, loose fit uniforms and black shoes, were heretofore taboo in the space of “traditional” NCAA basketball.

To truly understand the overall impact of the 1991–1992 University of Michigan Men's Basketball team, the athletic and sociological background of the Fab Five must be examined. The Fab Five consisted of Chris Webber, Juwan Howard, Jalen Rose, Jimmy King, and Ray Jackson; all rated as Top 100 college prospects as high school seniors. Four of the five, Webber, Rose, Howard, and King, were ranked within the top ten in the nation, and participated in the 1991 McDonald's All-American Game, the highest honor for high school players (see Appendix A). Beyond their athletic prowess, all five young men were Black from the urban inner city (1, 2). With the one-of-a-kind recruiting class and reigning most electrifying recruiting class in college basketball history (3), the Fab Five led the Michigan Wolverines to two-consecutive National Championship games in their first two seasons in Ann Arbor, Michigan (4). The 1991–92 season culminated in a runner-up finish to Duke University (71–51), and the 1992–93 season ended in defeat to the University of North Carolina (77–71).

Despite some seeing their lack of a National Championship as a shortcoming, the Michigan Fab Five's legacy bookmarks the nexus of the Black athlete experience in collegiate sport and cultural change in collegiate sport. To examine their cultural influence, we frame the collective identity of the Michigan Fab Five through their symbolic interaction while tracing the interest convergence of the Black athlete and National Collegiate Athletic Association. Denzin (5) explains SI as a study of the intersections of interaction, biography, and social structure in a particular historic moment. We explore this convergence through the study of the intersections of the interaction and biography of the Michigan Fab Five and the social structure of NCAA athletics as a specific historical moment.

## The starting five: significance of the five

Sport sociologists have critically studied constructs of the Black athlete experience (6–15), bridging the five legal-grounded tenets (16, 17) and education-grounded tenets (18) of critical race theory (19). The existence of the black athlete in NCAA sport has theoretically served as the vital symbol for epistemological and ontological premises that seek to theorize sport as a space. In college basketball, the construct of race and the ideology which produced racism created a perception of the capacity of the Black athlete. Historically, the recruitment of the Black athlete has been and continues to be a sociomoral issue in college sport. By the mid 1950s, northern colleges reached a token level of integration amongst their basketball and football programs, agreeing to ban Black athletes in competition against southern colleges (20).

Conjunctively, northern colleges willingness to recruit Black athletes and legislative changes associated with the United States Supreme Court's Brown vs. Board of Education desegregation ruling changed the political landscape of college athletics. Wayne State University (Mich.) Men's basketball became the first non-historically black college to play five Black athletes together on the court, beating major university programs such as DePaul, Detroit, Duquesne, Georgetown, Marquette, Memphis State, Niagara, Penn State, St. Francis (Pa.) and St. Mary's (Calif.) (21). In 1957–58 NCAA season, blacks accounted for five of the six NCAA consensus first-team All-Americans – University of Seattle's Elgin Baylor, Kansas State University's Bob Boozer, University of Kansas' Wilt Chamberlain, University of Cincinnati's Oscar Robertson and Temple University's Guy Rodgers (22). The 1958 NCAA basketball all-American selections created the first all-Black All-American team. Despite the acknowledgement of Black talent, massive resistance remained amongst predominantly White institutions in the south.

Against the sociopolitical background of college athletics in the south, Don Haskins led Texas Western basketball program highlighted the benefits of recruiting black athletes. During the 1965–1966 season, the Miners of Texas Western University experienced the most successful season of its history, losing only one regular season game. Its competitive success was notable, however the racial composition of the program's roster brought the most attention; consisting of seven African-Americans, four Anglo-Americans, and one Mexican American which contrasted its NCAA final opponent, the University of Kentucky's all-White roster. Prior to the 1966 NCAA national championship game in which Texas Western University started five Black athletes^1^, informal ethics limited coaches to playing three Black athletes at a time (23). For the first time ever in the NCAA final game, five Black starters played against five White starters.

Within the hegemonic structure of NCAA athletics, the Black athletes of Texas Western University presented a unique voice of color, from David Lattin's explosive slam dunks to Bobby Joe Hill's stylistic ball handling. The Michigan Fab Five amplified the voice of the Black athlete established by the Texas Western University athletes at the intersection of race and the socially constructed role of the NCAA “student-athlete”. As five “true” freshmen, the Michigan Fab Five's symbolic interactions challenged the dominant ideology of the Black athlete, centralized and publicized the experiential knowledge of the Black athlete, and highlighted diverse perspectives to advocate for structural change to the oppressive system of NCAA athletics. The catalytic significance of the Michigan Fab Five is centralized within the interest convergence of the NCAA athletics and the identity of the Black athlete.

## Symbol of the freshman sensation (hooper)

While the 1972 NCAA ruling that, “…granted freshmen eligibility in the two biggest team sports, thus making first-year students varsity candidates in all sports at all colleges,” was transformational (24), “Freshmen Given Varsity Status in Major Sport”) it was but a precursor to the impact of the Fab Five on collegiate sports. During the Fab Five era of NCAA Men's Basketball, the dominant Wolverines shocked the world with their youthful roster. Prior to their emergence, no other program, ever, had started five freshmen in a NCAA basketball game (1, 2, 4, 25). Retrospectively, the emergence of the Fab Five ignited the initial conversations of collegiate underclassmen departing from school prior to the exhaustion of their NCAA eligibility to enter the National Basketball Association. The physical talent, along with basketball IQ, made the five members of the Fab Five prominent candidates for the 27 NBA teams^2^ seeking to draft athletes onto their teams. With lucrative professional sport contracts and poverty-stricken families, the talent, popularity, anddemand for the of the Fab Five sparked the discussion of amateurism within NCAA athletics. These critical discussions on amateurism, eventually (almost a decade later) created what is now called the “one and done” rule – legislation implemented by the NBA marking eligibility to the NBA draft for all athletes 19 years of age and older. Furthermore, the five freshmen had vibrant personalities and became popular on the Michigan campus, as well as in the Sports Media. Under the guidance of Steve Fisher, the Fab Five infused the NCAA basketball world with their style, which included aggression, trash talk, and celebrations. Their ability to communicate both “on-and-off the court,” displayed their capacity to express their thoughts. Through the lens of symbolic interactionism (26), the Fab Five understood that their interactions amongst themselves and others allowed people to understand situations and reason through choices.

The Fab Five constructed their biography through their personal and collective observation of others, their life experiences, intrinsic reflection, and willingness to discuss with others. Within the Black athlete identity, the Fab Five developed a symbolic “self”. “Self” viewed by Blumer (26) as a uniquely human attribute developed through a continuous process of social interaction. As individuals, the Fab Five embraced the creative, unpredictable aspect of self, comfortable and reactive to the immediate situations. Collectively, the Fab Five established an organized set of attitudes that influenced their habitual action and conscious responsibility. Within the dynamic environment of NCAA sport, the Fab Five exhibited the ability to react creatively to the experience of the Black athlete while stabilizing the meanings formed and choices they made (5, 27). Evidence of the conceptualization and reconceptualization of the Michigan Fab Five can be found in their style of dress and play, their application of socialized ethics, and their existential activism for “student-athlete” empowerment. Many familiar with the basketball culture would call the conceptualized symbol birthed from the Fab Five genealogy a “hooper”. Markers of this genealogy can be seen in the physical appearance, style of play, and competitive character of the athlete. Prior to the Fab Five, the “hooper” was a social deviant in college basketball. Furthermore, the acceptance of a freshman “hooper” was an outlier; the moral atmosphere failed to ethically accept and support the ideal and quantity of “hooper(s)” at that juncture.

### Ethical invasion of the Fab Five

During the 1980s and into the 1990s, NCAA basketball players wore uniforms that could be considered uncomfortable, hideous in current fashion terms, and a form of indecent exposure when compared to contemporary styles. Jerseys were skintight and so snug that athletes could not wear a t-shirt underneath it. Athletic socks were white and pulled as high as possible, and simple, white and largely unadorned shoes served as the popular choice for major NCAA men's basketball programs. Along with the uniforms, most NCAA men's basketball players were clean cut in terms of appearance. Standard haircuts and clean-shaven faces were the norm, while tattoos and other fashion statements were frowned upon, as they were thought to be individualistic and self-serving, which coaches and administrators believed would shift the focus away from the performance of the team. Unlike the conservative culture of NCAA basketball, the Fab Five defied all stylistic and apparel expectations. Groomed with bald heads, tattoos, long and baggy shorts, loose fitting jerseys, with black shoes and socks, the Fab Five invaded the buttoned-up culture of the sport (1, 25).

The vivid fashion of the Fab Five was amplified by the style of basketball exhibited by five young athletes. Aggressive slam dunks were highlighted by difficult and fancy ball handling, all of which were deemed unnecessary and unsportsmanlike by basketball critics, experts, media, and the Fab Five opponents. The Fab Five's flashy bravado on-and-off the basketball court was perceived by most as a false sense of confidence and a sign of disrespect to their opponent, as well as the game of basketball (1, 2, 25). Further antagonizing their critics was the Fab Five's use of trash talk, a term with which they were not familiar.

Often accompanied with demonstrative behavior, the Fab Five enjoyed and thrived during competition that involved constant verbal chatter amongst themselves and towards their opponents and their fans (Appendix B). Though the University of Michigan Men's Basketball team did not garnish a reputation for flagrant or technical fouls, nor cheating, the verbal chatter during competition was translated by spectators, and many of the NCAA board members, as verbal assault, which violated everything about sport. The antics of the Fab Five were perceived as blatant disrespect to the morals and history of sport, specifically, respect, honesty, and beneficence (28). This perception placed a divisive narrative between the youthful nature of the Fab Five, and the aggressive way they played, with what was deemed to be (historical) appropriate behavior of sportsmen. Mistakenly, critics' observations were analyzed through a dogmatic, authoritarian lens, which disregarded the benefits of street-style of play, and strategically (and aesthetically) enhanced their team's success on the court.

Often with great charisma and exuberance, the Fab Five verbally interacted with themselves and opponents at the same pace as their attacking offense. To administrators, officials, and other coaches in the NCAA, the interactions were intended to antagonize and disrupt the focus of their opponents. Based on this interpretation, the NCAA chose to act to limit and regulate the types of personal interaction during competition, creating additional guidelines to which such behavior would be penalized with technical fouls (2).

With the intention of protecting the values associated with sport, such as respect, courage, and honesty, new sportsmanship rules were established and enforced immediately. As expected, the immediate effect of the sportsmanship rules stifled behavior of other teams, who sought to mimic the behavior (and success) of the Fab Five. The effect of the new rule changes on sportsmanship pleased the rule-makers, and stakeholders, as a sport modeled after the English protocol, which ensured the comfort of the upper-class society, or elite class. In a basketball community, which still served White America due to the social temperament of American society, these rules were supported and accepted without any consideration for the culture of the violators (e.g., the Fab Five). In an analysis of the Black male baller C. Keith Harrison et al. (8) found themes: (a) trapped, (b) against the world, (c) Streetz R Death, and (d) Ambitionz when contextualizing hip hop lyrics and the experience of the Black athlete in college sport. Aligned with these themes, the behavior of the Michigan Fab Five gave meaning to their ambitions of winning and advancing to the NBA against the odds as a Black athlete. Furthermore, the Fab Five were not able to respond without thought to situations but as an “acting organism” who had to forge and direct their line of action (26).

### Cultural artifacts of the Fab Five

It has now been more than 30 years since the Fab Five shocked the world, with their style of play, and their perceived arrogance. Their sociocultural effect aligned the sociocultural values of their community, within the game of basketball. Such alignment influenced American society and the culture of basketball, simultaneously. The baggy uniforms, the tattoos, the bald heads, the black shoes and socks, and the public's gravitation to the hip hop music genre, prevailed. The occurrences of extracurricular verbal and physical interactions, between teammates and opponents, became more prevalent during competition. As Black athletes who displayed the capacity to compete on an elite level, with an ethical standard that signified upon and defied the racial constructs of the dominant ideology of the Black athlete, the sociocultural effects of the Michigan Fab Five can be seen today through (1) the amplified voice of the Black athlete i.e., Angel Reese of Louisiana State University, through competitive play and sport media, (2) a clearer view of distributive justice regarding the commodification of the likeness of the college athlete through Name, Image, Likeness legislation, (3) the cultural approach to the approach to restrictive measures of playing time based on age, and (4) the interactive manner in which the game is played on all levels. Most importantly, it is critical to note that members across all sociological demographics within the basketball community, have been influenced by their style, actions (on-and-off court), and authentic cool pose. The reflexive action of “turning back experience upon self” reformulated the social process into the experience of the athlete in NCAA basketball; The Fab Five's experience can be articulated and shared with others in this manner (27).

### Sociocultural clash between tradition and the Fab Five

In modern American basketball, competition figuratively serves as a battlefield for the clash of cultures in addition to the passionate competition between groups of competitors. This clash is between opponents with different cultural backgrounds. As a big-ticket revenue sport, basketball is dominated by African American athletes. Therefore, the sociomoral conflict within basketball pits the African American athletes against the rules of sport which are grounded by white America culturally. Critically, it is important to note that the sociomoral constructs of basketball must consider the intercentricity of race, racism, and classism (18).

The behaviors and attitudes of white America often clash with the adaptive patterns of behavior of those in black America (29). Acts by black people are misunderstood and reprimanded because they are not quite right, not quite “white” (30, 31). Coolness and stylistic improvisation in speech or movement, among other black masculine response to harsh living conditions, have been misinterpreted in classrooms, the work place, and the sporting field (22, 32–34). A subject in a study administered by Andrews [(35), p. 77], provides supportive testimony regarding discourse in sport that has been interpreted as bad behavior:

*The majority of black kids that are playing are from the inner cities, and I think that expression comes from maybe some of the things that they've experienced. Maybe they're a little more happy to be out of the situation than other kids are…I'm away from the violence, and I don't have to worry about somebody popping their cap [gun] in me. So maybe I'm a little happier when something goes right for me on the field, so that causes me to express myself more than others…You've come a little longer way than someone else*.

In the same study (35), a subject cites aggression as a primary way of white expression and physical talent a key factor in the black movement in sport.

*You can separate the black athletes from the white athletes as far as their mentality and their emotions…it's hard to explain…it's just the physical appearance and the way they move…it's the style, I'll put it like that. It's the style …the black athletes have a lot of aggression…and that's what separates them*.

Majors and Billson (36) attribute the priority on style to the Cool Pose, a carefully crafted persona based on power and control over what the black male says and does, how he plays his role. For black males, who have limited control or access to conventional power or resources, cool pose is empowering (36). Power over one's self is the most important form of power, particularly in an environment where manipulation and control over others have been raised to the level of a fine art, where contest and game playing are often the rule, not the exception (29). In an interview leading into their first nationally-televised contest vs. the number one ranked, Duke Blue Devils, the five freshmen displayed their cool pose while attempting the repossess their power. Concluding the interview, the five simultaneously state, “Five Times! No Fabulous Five, just Five Times!”, followed by Jalen Rose proclaiming “What up Detroit!…I know I need a fade, can't get a fade every day” (2). Leveraging the platform, the young men were confident in manipulating their narrative within a basketball culture that favored social order and perceived conformity. Within this social anomaly, the five freshmen attracted social attention and critique with their authentic charisma and transparency individually and collectively.

Many African American males have a knowledge of expression that is often peppered with verve, rhythm, improvisation and individualistic style, even within the context of team (31, 33). The variation of the extent of this knowledge differs between each individual, for some not allowing as much celebration or individuality in a team context. A knowledge often struggles to repress, evade or deligitimate other knowledges (34). In sport, a macro-social culture, knowledge serves the cultural interests, both materially and politically, of the social formation that produces knowledge, and resulting the effectiveness is likely in direct proportion to the power of the interests behind the knowledge (32). Power, then, produces a knowledge which is disguised as truth (37). The truths struggle to repress, evade or deligitimate other knowledges (32).

The sociocultural dynamic of American society mirrors the continuing conflicts between white and black America, often focused on expressive behavior such as clothing, hair styles, facial hair, speaking styles, enunciation, and even the loudness of African Americans. The truths of college basketball culture in the 1990's struggled to deligitimate the knowledge of the Fab Five. Moral commentary produces discourse in action between knowledge and truth. Prefacing the 1993 NCAA Men's Basketball Tournament, television analyst led the discourse stating, “Michigan has the most overrated group of underachieving teams of all time… They come in strutting like, they think they are better than they are” (2). During the post-game highlights following the University of Michigan's Men's basketball team's second consecutive loss in the NCAA championship game, Keith Olberman (38) states:

*Webber failed to remember his team had no time-outs remaining and was thus penalized with a technical foul, thus losing the ball and the game by four points. Michigan played all this year with that in-your-face style and they got caught on a little fundamental. It's kind of a morality play, if you believe in that sort of thing*.

Olberman's (38) remarks essentially states that bad behavior is punished with bad results. No bad individual deed or team goes unpunished – morality takes care of that. In the same segment, Bill Walton proclaimed, “They (Michigan Fab Five) are the epitome of what is wrong with basketball players” (2).

Race and culture often serve as the foundation for the awareness of social phenomena. Sports discourse, like discourse in general, is never neutral of objectivity and is often rooted in essenstialism. Production and repression through sports discourse is always politically active in specific social conditions, becoming a terrain of struggle – discourse is always a matter of contestation (34). Knowledge is activated socially through discourse and discourse circulates knowledge and carries its power into a variety of social situations. Sports media discourse is typically allied with those in power whom attempt to control the sport in whatever way they choose. The Fab Five's existential being defied the essence of an NCAA college basketball player. The discourse which engulfed the Fab Five was a product of the clash between the knowledge of the White-clad tradition of college basketball and the knowledge of the Fab Five- an evolutionary clash for social power. This discourse must be assessed in considering Fab Five's influence on the culture of basketball and American culture.

## Power of the Fab Five

To conceptualize the influence of the Fab Five, we further explore the nature of their knowledge of expression and how it is attached to power. The term knowledge can be used in a plural sense as well, intended to be literal and figurative-actionable and symbolic. A knowledge of expression is a specific way of looking at expression, a perspective on expression helped by an individual or many. Knowledge of expression is the many specific ways of looking at expression taken together. The Fab Five pioneered foundational knowledges of expression, expressed in variety of ways – smack talk, basketball fashion, hip hop, and social resistance through excellence. The social context for interaction is society and how society develops as a result of the interwoven patterns of interaction and action (27).

### Smack talk- competitive language of hoops

Coupled with their dominant basketball performance during their tenure at the University of Michigan were their jubilant, yet demonstrative interactions amongst themselves and opponents. Coined as trash talk (39), and interpreted as physical and psychological intimidation (28, 40), the behavior of the Fab Five was categorically interpreted as incivility. Yip et al. (41) describes trash talk as incivility expressed in a competitive context in which two or more parties are vying for resources, recognition, or status. In a study accessing the intentionality of trash talk, Fennell (42) found that male and female basketball athletes, along with football athletes, high school level and above, significantly exhibit such behavior with positive intentions.

Such intentions positively influence relationships between peers and opponents, grounded by the conceptual cooperation required within competition. Additionally, athletes use such interactions to ethically gauge those they signify upon to protect the moral community of basketball which solely values the membership of the athlete.

Considering the moral reasoning incorporated with the use of competitive language, the interactions of the Fab Five can be interpreted as smack talk (43). Most known for his smack talk towards Duke University's Christian Laettner, Jalen Rose called Laettner “an overrated (expletive) until I saw him ‘ball’” Aligned with the ethical double-voice of smack talk, his smack talk was also aimed at his teammates. For example, challenging his close friend and fellow Fab Five member, Chris Webber, Jalen states, “Chris, you a beast! Dunk on all of them!” Fellow Fab Five member, Ray Jackson stated that (2). Moreover, the Fab Five applied their smack talk towards their upperclassmen teammates in an informal scrimmage, during their first day on campus; the event shook the internal world of Michigan basketball as the then “five freshmen” affirmed their smack talk (25). Rather than aligning with the interpretation of trash talk, the smack talk of the Fab Five sought to establish social justice within the competitive environment. Furthermore, the once offensive behavior, publicly pioneered by the Fab Five has now become the norm of college basketball players, White and Black.

Analyzing smack talk as a knowledge of expression sociologically constructs what some may consider a player's only code. Much like psychologically foundations of the urban game, The Dozens, used by African Americans practiced in segregated America, the player's code of conduct empowers athletes within the psychological warfare of competition and the commercialization within it. A portion of this code provides leeway for pure excitement and joy. Appealing to the ethos of sport, the athlete community itself accepts it as a strategic ploy that does not convey the insulting message that it carries in other contexts. Additionally, smack talk is justified because it is not only consistent with, but actually enhances one of the main goals of athletic competition: testing athletic excellence (39). The Fab Fave used smack talk to establish their cool pose and insulate their competitive environment. The strategical aspect of competitive sport places heightened importance on the importance of talk on the act of competing. The athlete who struggles to maintain their composure usually fails to achieve victory. Competitive sport, which is the ethical guideline within the moral community, allows smack talk to serve as a comradery builder. Competitively, smack talk brought the Fab Five together. The resiliency built through vulnerability in sport produces the strength uphold one's character as an athlete. Within the moral construct, smack talk required each member of the Fab Five to maintain their integrity. Socially, smack talk ethically challenged all participants within the competition while eliminating any influence of sociological constructs and intersections. The competitive language of the Fab Five challenged the competitive character of their teammates and opponents as individuals. More significantly, the cultural impact of the Fab Five challenged and changed the ethos of college basketball indefinitely. Their legacy empowered all athletes to conceptualize themselves individually while enabling the collective to reconceptualize itself through performance within social conflict – a lesson on power and socialized ethics, perhaps society may learnfrom sport.

### Hip Hop- the soundtrack of hoops

Accompanying the Fab Five's knowledge of expression, their love for the music genre, hip hop artistically narrated the ontological perspective of the college “hooper” and perhaps many of their peers. Much like the “hooper” identity, hip hop music authentically integrated itself into American society with fashion, bravado, and intent to hold power within a transactional society. Culturally popular in Black America, the Fab Five became the symbolic ambassador to the cultural framework associated with hip hop. Prior to the arrival of Fab Five on the national stage, sport media exhibited little interest into the pregame entertainment of competing athletes. With growing popularity surrounding the group of athletes, sport media began to inquire about the choices of music playing through their mobile headphones as they entered arenas. Recorded and depicted in the ESPN 30 for 30 film (2), the Fab Five unapologetically shared their favorite artists including rap groups such as N***** With Attitude (NWA), EPMD, and Das EFX. You could hear EPMD booming in the Michigan locker room or see the players jump on the scorer's table and wave their arms like in Naughty By Nature's “Hip Hop Hooray” video after a victory (44). Much like the response to their smack talk, the basketball culture along with American society associated the Fab Five's impact on the culture through the vernacular traditions of the artists of their choice. Such association grew to categorically identify the style of play initially embraced by the Fab Five as “hip hop ball”. Hip hop also contributes to representations and performances of identity, often in ways that cut against the stereotypical grain (7). Furthermore, the style of play opposing the traditional style of play can be traced to the Fab Five.

Hip hop ball is defined as the combination of basketball with hip-hop, two cultural practices involving young Afro-Americans from popular backgrounds (45). Opposing traditional styles of playing, hip hop ball is considered a basketball practice characterized by a more spectacular style, putting a stronger emphasis on individual skills (46, 47). Axthelm (48) coined the style of play as “The City Game.” Unique basketball fashion and specific musical preferences not only served as knowledges of expression for those that identified with sociocultural constructs but served as mechanisms of protest within sociopolitical struggle. Basketball, inside a society structured by racism, is an institution central to the battle of African Americans for equal civic rights. Therefore, basketball serves a social space of cultural creativity and symbolic recognition, of political resistance and of civic reclamation for part of the black community (49).

Through the game of basketball, the Fab Five served as symbols of civic reclamation, shifting the social power bloc socioeconomically. The basketball fashion and ability to signify upon the culture through smack talk and hip hop enabled not only the Fab Five to earn recognition for their political resistance but for all Black basketball players. Such influence is evident on all levels of basketball, most glaringly in the National Basketball Association. Athletes such as Allen Iverson, Carmelo Anthony, and Stephen Marbury were labeled as disrespectful players, raised by hip-hop and intentionally targeted as cancers to the profitability of the professional league. Within the same breath, corporate brands like Reebok and Nike commodified the rebellious the acts. Given the moniker, “The Answer,” Allen Iverson was positioned by Reebok as the rebel within the hip hop tradition, who symbolically is the answer to the socialization question within the league.

At the intersection of hip hop and sport, Reebok's branding of Allen Iverson was an indicator of the tanning of America (50). Stoute (50), an American record executive, describes tanning as the process in which hip hop created a culture that transformed the American economy (51). Prior to the branding of “The Answer”, Nike commodified the likeness of the Michigan Fab Five in the same manner, the first of its kind for college sports programs. In 1992, Nike marketed the release of all black Nike Air Force 180 Max basketball shoe as the “Fab Five”; and again in 2004 with the re-release of the Fab Five Huarache 2k4 basketball shoe, a shoe the Fab Five wore in the 1992 NCAA tournament as cited by Sports Business Journal (52). Culturally, the Fab Five Huarache 2k4 basketball shoe is iconic, fashionable, and historical grails -artifacts of the tanning of college basketball.

## The tanning of college basketball

The revolution of basketball culture metaphorically resembles the aesthetical interpretation of the physiological response to a moderate puncture wound to the skin; The Fab Five sharply and quickly breached a tightly secured college basketball culture. The adrenaline from witnessing the high-flying, gregarious young men produced a numbness, presented by an ignorant neglect for the athlete experience. Following the intial adrenaline rush, the gatekeepers of the culture- sport media and members of sport governance bodies sent signals to alarm the culture of the Fab Five's presence. Its initial response was to temper the Fab Five's influence by controlling the narratives and boundaries in which the Fab Five existed. Through NCAA amateurism and sportsmanship legislation, the basketball culture began its recovery from the dramatic entry of the Fab Five as they matriculated to the NBA. College basketball culture began to rebuild their culture under the foundational constructs of sportsmanship and amatuerism; Revisiting the puncture of the Fab Five, the NCAA handed down sanctions to the University of Michigan Men's basketball program resulting from the Ed Martin Scandal^3^.

The results of the Ed Martin sanctions could have been the preverbial bandaid that was intended to ensure the recovery from the exposure from the Fab Five.

Though there has not been another recruiting class like the Fab Five in college basketball, recruiting Black athletes, having multiple true freshmen in the starting lineup, and entering the NBA draft prior to exhausting NCAA eligibility are the norm in college basketball. In fact, the 2013–2014 University of Kentucky Wildcats are the only other college program to start five true freshmen in a competition (54). Historically, the 2014 Wildcats are a significant product of the Fab Five legacy. Like the Fab Five, the Wildcats- Aaron and Andrew Harrison, James Young, Julius Randle, and Dakari Johnson were highly recruited, McDonald's All-Americans, and African American. Symbolically more significant, they played for the program which sociopolitically and competitively opposed the Miners of Texes Western University in the 1966 NCAA national championship. True convergence of interests of university stakeholders and potential college athletes, exposed by the publically unapologetic and symbolic entity of the fab five has obliterated the white pillars of even the southern “blue blood” universities.

According to NCAA (55) Demographics Database, 55 percent of Division I Men's Basketball athletes report their race as Black, while 22 percent report as other. The Fab Five not only served as the sharp instrument to amplify the college basketball culture for Black America but also as the pioneering activists for college athletes' rights. The commodification of all things Fab Five placed a bright light on the young Black men, producing profit for the University of Michigan and its corporate partners while witholding compensation to the Fab Five. The branding of the Fab Five serves as a powerful foundational casestudy for the need for Name, Image, and Likeness legislation in American college sport. Exhibiting their cultural maturity, the Fab Five verbally and non-verbally acknowledged and protested the oppressive capitalization of their likeness (1, 2). Retrospectively discussing the Fab Five's experience in college basketball, Ray Jackson (3) states, “We got to college and started understanding the hypocrisy in the game, with the schools making millions and us sitting around poor as hell… We wanted to change the dynamics of that, get the athletes feeling empowered a little more. The Fab Five empowered athletes far beyond what they could have imagined at that juncture. College athletes now have the opportunity in which the Fab Five yearned for as college athletes. Critically analyzed through the lens of interest convergence (16), the exploitative capitalistic treatment of the Fab Five socioculturally drove racial progress for racially minoritized individuals in NCAA sport – however, only when the institution of the NCAA, led by the White power bloc's interests of capitalism was served. The hypocrisy has now been acknowledged by the institution of the NCAA. In June 2021, the NCAA (56) adopted Name, Image, and Likeness (NIL) policies allowing college athletes to benefit from name, image, and likeness opportunities. The legislation and subsequent calls for reform in the form of class action and other lawsuits, ultimately reframed the concept of amateurism in competitive sport. All states within the United States have passed legislation outlining the rights to NIL opportunities (57). This structural and legislative change has required universities to evolve how they approach organizational strategies and resource acquisition. Intended to attract the most talented prospective athtletes, university athletic departments have partnered with third-party NIL collectives to accrue and distribute NIL resources. The current trend of NIL collectives, pioneered by the Gator Collective (58) (University of Florida) and followed by the likes of Rising Spear (59) (Florida State University) and 12th Man+Fund (60) (Texas A&M University), has conceptually eliminated amateurism and created a “pay-for-play” system. Spotlighting the lucrative business of NIL in college basketball, Louisiana State University Women's Basketball star, Angel Reese (61), when asked about her intent to forego her NCAA eligibility and pursue a career in the Women's National Basketball League stated, “I’m in no rush to go to the league… The money I’m making is more than some of the people that are in the league that might be top players”.

The landscape of college basketball culture has experienced a transformation. The willingness to be more athlete-centered on the organizational level has transformed attitudes and beliefs around topics of equity and discrimination. This transformation was accelerated when University of Michigan's Steve Fisher chose to enable the Fab Five to authentically exist within a static culture. At the time of their collective appearance, the Fab Five turly disrupted basketball culture on all levels.

The changed dynamic in college basketball culture did not remain on the college level. Due to the performance and entertainment demand within the capitalistic environment of NBA organizations, young and demonstrative athletes have become high demand in the league. In a study assessing sport analytics in the NBA, using player efficiency rating [PER] and value over replacement player [VORP] statistics, Zestcott et al. (62) found that players with less college experience had better offensive, defensive, and advanced metrics, despite making more mistakes and committing more turnovers. Across all levels of basketball, the fast-pacced playground style of basketball permeates through the minds of coaches and athletes alike; The very same style of play which was categorized in association with the perceived deviant Fab Five (1).

The trace of the Fab Five is a permanent cultural marker within the basketball culture. Subjectively, critics may reflect upon the visceral impact of the Michigan Fab Five, while the current athletes view the imperfect deviance definance as a necessary hardship which produced strength and freedom. The Fab Five served as the cultural pacemaker to contemporary American basketball culture and NCAA athletics, by challenging the culturally insensitive ideology of college sport while exposing the unbalanced distributive justice within it. As a result, college basketball has a new culture. The personal identity of the athlete has been embraced. The moral judgment and action of structural entities and actors are centered around the socialized ethics of the collective athlete population. Acknowledging the capitalistic and exploitative nature of college basketball, progress towards socioeconomic mobility of college athletes has been made through legislative and practical changes. This is a paradigm shift realistically enabled sport to truly become a microcosm of American society, highlighting sociological intersections within sport, further illuminating the athlete population as a special population. Any anthropological examination of the contemporary college athlete cannot disregard and shall consider the ecological impact of the Michigan Fab Five.

## Data Availability

The original contributions presented in the study are included in the article/Supplementary Material, further inquiries can be directed to the corresponding author.

## Footnotes

^1^Four of the The 1966 NCAA University Division Basketball championship was a contest between the Texas Western University Miners coached by Don Haskins and the University of Kentucky Wildcats coached by Adolph Rupp. Haskins started 5 Black athletes in the contest. Haskins’ roster defied the racial norms of the sport with 7 Black athletes and 5 White athletes. The Minors finished the season as national champions losing only one contest.

^2^Four of the five members were drafted into the NBA. Webber, drafted No. 1 overall in the 1993 NBA draft, played 15 seasons in the league. Webber was a five-time NBA All-Star, the 1994 NBA Rookie of the Year, and had his No. 4 jersey retired by the Sacramento Kings. Rose, drafted No. 14 overall in the 1994 NBA draft, played 13 seasons in the league and currently serves as an NBA analyst for ESPN. Howard, drafted No. 5 overall in the 1994 NBA draft, played 19 seasons, winning two NBA championships. Howard currently as the Head Coach of the Men's Basketball program at the Fab Five's alma mater, University of Michigan. King, drafted No. 35 overall in the 1995 NBA draft, played two seasons in the league. Jackson, the lone member to never play in the NBA, was named the CBA Rookie of the year in 1996.

^3^In the late 1990's the NCAA investigated the University of Michigan's Men's basketball program. The NCAA found that University of Michigan booster, Ed Martin provided impermissable benefits to members of the program, including Fab Five's Chris Webber. As a result of the investigation, Michigan was required to vacate all wins associated with athletes corroborated within the investigation, including during the Fab Five era. Additionally, the basketball program was placed on four years of probation, four years of postseason eligibility and lost one scholarship each year for the four-year probationary period. The NCAA also required the University of Michigan to distance itself from those who were found guilty of taking payments, leading Webber to be banned from any association with the University until 2013 (53).

## Appendix A

The members of the Michigan Fab Five (L to R): Jimmy King, Juwan Howard, Chris Webber, Jalen Rose, and Ray Jackson.

## Appendix B

Fab Five's Jalen Rose challenges Chris Webber during 1992-1993 competition.
